# Enhanced tolerance of transgenic potato plants expressing choline oxidase in chloroplasts against water stress

**DOI:** 10.1186/1999-3110-54-30

**Published:** 2013-09-03

**Authors:** Yu-Jie Cheng, Xi-Ping Deng, Sang-Soo Kwak, Wei Chen, Anthony E Eneji

**Affiliations:** 1 State Key Laboratory of Soil Erosion and Dryland Farming on the Loess Plateau, Northwest A&F University, Yangling, Shaanxi, 712100 China; 2 Department of Forest, Northwest A&F University, Yangling, Shaanxi, 712100 China; 3 Enviromental Biotechnology Research Center, Korea Research Institute of Bioscience and Biotechnology, Daejeon, 305-806 South Korea; 4 Department of Soil Science, University of Calabar, Calabar, Nigeria

**Keywords:** Choline oxidase, Glycine betaine, Re-watering, Transgenic potato, Water stress

## Abstract

**Background:**

Glycinebetaine, whose biosynthesis could be catalyzed by choline oxidase (COD), is an extremely efficient compatible solute for scavenging oxidative stress-inducing molecules and protecting the photosynthetic system in plants. To study the effects of the codA transgene for choline oxidase on the drought resistance and recovery, a transgenic potato cultivar (SC) bearing *codA* gene and a non-transgenic (NT) control cultivar were raised in pots under moderate and severe drought stress. The experiment was constituted by a two-day-pretreatment with 20% PEG and a four-day-water stress combined with two-day-recovery treatment.

**Results:**

Under the four-day-water stress, plants were provided with normal water condition, 10% or 20% polyethylene glycol. The results of pretreatment showed an expression of *codA* gene in transgenic potato and an accumulation of glycine betaine (GB); leaf water potential was higher in SC than in NT. In the stress-recovery-treatment, SC showed stronger antioxidant ability, more efficient photosynthetic system, higher chlorophyll content, lower malondialdehyde content and better recovery from water deficit stress than NT.

**Conclusion:**

Although this work concentrated on the short-term water stress and recover treatments on transgenic potato plants with the over-expression of *CodA* gene and its control line. The datas shows that the exogenous *codA* gene provided potato a stronger drought resistance and recovery ability.

**Electronic supplementary material:**

The online version of this article (doi:10.1186/1999-3110-54-30) contains supplementary material, which is available to authorized users.

## Background

Glycinebetaine (GB, *N,N,N-trimetrimethylglycine*; hereafter betain) is a quaternary ammonium compound that occurs naturally in a wide variety of plants, animals and microorganisms. The accumulation of GB is induced and synthesized in the chloroplasts of higher plants under various abiotic stress, such as high salt, drought and cold (Jagendorf and Takabe 2001; Rontein et al. 2002), and the exogenous GB could enhance the resistance ability to drought (Mahouachi et al. 2012). Glycinebetain affords osmoprotection for plants and protects cell components from harsh conditions by functioning as a molecular chaperone (Sakamoto and Murata 2002).

Furthermore, it could stabilize the higher-order structure of protein and protect the activities of intracellular protein and metabolic enzymes (Demiral and Turkan 2004). In photosynthetic systems, GB efficiently protects various components of the photosynthetic machinery, such as rubulose-1,5-bisphosphate carboxylase/oxygenase (Rubisco) and the oxygen-evolving photosystemII (PSII) complex from stress (Murata et al. 2007). It preserves the normal cellular turgor pressure, playing an important role in respiration and photosynthesis. An exogenous application of GB improves the growth and survival of a wide variety of plants under various stress conditions (Ashraf and Foolad 2007; Hoque et al. 2007; Park et al. 2006; Chen and Murata 2008). The fact that many agronomically important crops, such as rice and potato, are betain-deficient has inevitably led to proposals that it might be possible to increase stress tolerance by genetic manipulation that would allow non-accumulators or low-level accumulators to accumulate betain at protective levels (McCue and Hanson 1990).

Glycinebetaine has three main synthetic pathways in different organisms (Sakamoto and Murata 2000). Therefore, different methods could be used to introduce a GB synthetic system into non-GB-accumulating plants to improve their stress tolerance. One of the methods was the introduction of the BADH (betaine aldehyde dehydrogenase) gene, which has been frequently introduced into a variety plants including tomato (Jia et al. 2002), tobacco (Yang et al. 2005; Ci et al. 2007; Zhou et al. 2008), wheat (Guo et al. 2000) and potato (Zhang et al. 2009) for enhanced tolerance of salt, drought or extreme temperatures. The other was COD (choline oxidase), which itself does not exist in higher plants at all. Previous reports showed that the COD gene was also introduced into Arabidopsis (Sulpice et al. 2003; Waditee et al. 2005), tobacco (Huang et al. 2000), rice (Konstantinova et al. 2002; Mohanty et al. 2002; Kathuria et al. 2009), tomato (Goel et al. 2011; PARK et al. 2007; Park et al. 2004; Li et al. 2011), maize (Quan et al. 2004), potato (Ahmad et al. 2008) and *Eucalyptus globulus* (Matsunaga et al. 2012) to improve their stress tolerance.

Potato (*Solanum tuberosum*) is one of the leading crops throughout the world. It is cultivated in more than one hundred countries and regions, with total yield and cultivated area ranked fourth among crops, only after wheat, rice and maize (Jackson 1999). With the rapid economic development in recent years, potato is increasingly becoming an important cash crop and the potato industry has seen strong development recently. Hence, an excellent potato variety with resistance or tolerance of abiotic stress is required for the steady development of the potato industry (Jiang et al. 2008).

Different types of promoters have been used in plant transformation research; these promoters can be divided into three classes: constitutive promoter, organ-specific promoter and inducible promoter (Potenza et al. 2004). The most representative constitutive promoter is CaMV 35S (Odell et al. 1985), which is one of the most widely used promoters. However, the constitutive promoter might result in the over-expression of exogenous gene and break the regular growth process of plants (Scheid et al. 2002). The use of organ-specific promoters, such as potato tuber-specific patatin promoter, can make up for this weakness. SWPA2 (Oxidative stress-inducible peroxidase promoter) is inducible promoter cloned by Kim in 2003 from sweet potato. This was confirmed by transforming the glucuronidase gene with SWPA2 and CaMV35S promoters respectively into tobacco. Under water stress, the gene expression of SWPA2-*GUS* was 30-fold that of CaMV35S-*GUS*, suggesting that SWPA2 has a very strong stress-inducible ability (Kim et al. 2003).

In this experiment, we aimed to determine the influence of an introduced *codA* gene on transgenic potato under water stress and rewatering treatment, and provide a basis for research on new potato varieties and glycinebetain.

## Methods

### Materials

**Figure 1:**
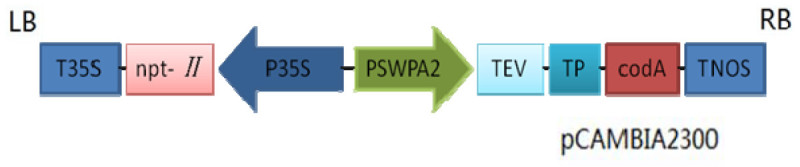
**Vector structure of pCAMBIA2300 with choline oxidase gene.** LB: left border; T35S: *CaMV35S* terminator; P35S: *CaMV35S* promoter; PSWPA2: *SWPA2* promoter; TEV: tobacco etch virus 5′-UTR; TP: chloroplast transit peptide; TNOS: nopaline synthesis terminator; RB: right border.

We used potted transgenic potato plants (SC) expressing *codA* gene (from *A. globiformis*) in chloroplasts under the control of an oxidative stress-inducible SWPA2 promoter (Kim et al. 2003) and non-transgenic (NT) control plants (*Solanum tuberosum* L. cv. Superior) (Ahmad et al.^2008^). The vector structure with *codA* gene is shown in (Figure 1). This experiment was divided into pretreatment and stress-rehydration-treatment.

### Pretreatment

Five four-week-old plants respectively from SC and NT were transferred to buckets filled with daily-aerated Hoagland nutrient solution. One week later, all plants were subjected to drought stress simulated with PEG6000 (polyethylene glycol 20%) treatment for 48 h. The fourth and fifth leaves were sampled from each plant at 0 h and 48 h after stress to determine the GB content, leaf water potential and expression of *codA* gene.

### Stress-rehydration-treatment

A total of 90 pots (45 for SC and 45 for NT) were used for this treatment. The two potato types were allocated to three drought treatments of no stress - normal water condition, PEG 10% (moderate stress) and PEG 20% (severe stress); each treatment has 15 replications arranged into a completely randomized design. Nutrient solution (with or without PEG) was changed at 9 a.m. each day just before the determination of photosynthetic activity. The stress continued for four days and then in the following two days all plants were provided with normal water condition. Five leaves (the fourth or the fifth leaf from each plant) from five plants were chosen randomly from each treatment everyday at 10 a.m. All the samples were immediately frozen in liquid nitrogen and stored at -80°C until required for analysis.

### Growth conditions

Each pot contained 1 kg disinfected dry vermiculite which was then watered to 70% of maximum field moisture capacity with Hoagland nutrient solution, with only one seedling cultivated in each pot. Pots were placed in a growth chamber under a 16 h photoperiod with light intensity of 300 μmol photons m^-2^s^-1^, 60% (w/v) relative humidity at day 25°C / night 20°C. Soil moisture was controlled by weighing each pot during the growth period.

### PCR analysis

Total genomic DNA was extracted from transgenic potato and control plant with DNA kits bought from Beijing TaiKe Biotechnology Limited. First-strand cDNA synthesis was performed in a 20 ul reaction mixture containing 1 ul of total plasmid DNA. The PCR was conducted with 0.5 ul first-strand cDNA with the primers of 5′-GCT GCT GGA ATC GGG ATA-3′(forward) and 5′-TGG GCT TAT CGC GGA AGT-3′(reverse). The amplification reactions occurred at 94°C for 5 min, followed by 30 cycles (94°C 30 s, 62°C 30 s and 72°C 1 min) and finally an extension cycle of 10 min at 72°C. The PCR products were separated on 1% agarose gel, stained with ethidium bromide, and visualized under UV. The expected size of the PCR fragment was 450 bp. The UV transilluminator was obtained from Thermo Company (USA), dNTP from Roche Company (Sweden) and *Taq* polymerase from Fermentas Company (USA).

### Glycinebetain content

Glycinebetain was measured via UV–VIS spectrophotometry (Huang et al. 2009). In brief, GB reacts with Reinecke’s salt under acidic condition to produce sediment of Reinecke’s salt, which was then dissolved in 700 ml/L acetone until the color turned pink. Acetone was used as blank control to produce an absorbance standard curve under 525 nm and the standard curve was used to determine the GB contents in samples.

### Photosynthetic system and leaf water potential

From 9 a.m.—10 a.m. each day, the fifth mature and well-exposed leaves from top in five randomly tagged plants in each treatment group were sampled for measurement with a portable photosynthetic meter, LI-6400; leaf water potential was determined according to the method of Turner using a pressure chamber (Turner 1988).

### Chlorophyll content

Mature and well-exposed leaves from plant (0.5 g fresh weight) were homogenized in a mortar and pestle using 10 ml of chilled 80% acetone. The homogenate was centrifuged at 10,000 rpm at 4°C for 10 min. The absorbance of the supernatant was measured at 646, 663 and 750 nm, respectively, and chlorophyll content was calculated as per the method of Arnon et al. (Arnon et al. 1974).

### Extraction and assays of the activities of reactive oxygen-scavenging enzymes

The determination of the activities of catalase (CAT), superoxide dismutase (SOD) and peroxidase (POD) followed those reported in Lee et al. (Lee and Lee 2002). Leaf samples (0.5 g) were homogenized in 8 ml of 50 mM potassium phosphate buffer (pH 7.0) that contained 1 mM EDTA, 1 mM ascorbic acid (ASA), 1 mM dithiothreitol (DTT), 1 mM L-glutathione (GSH) and 5 mM MgCl2. After sufficient grinding with little quartz sand, the homogenate was centrifuged at 20,000 rmp for 15 min at 4°C. The resultant supernatant was deep-freezed under -80°C and used for assays of enzymatic activity. Total protein concentration was determined according to the Bradford method (Bradford 1976) using the Bio-Rad protein assay reagent.

The activity of superoxide dismutase (SOD) was measured according to McCord and Fridovich (1969) with slight modification, by immediately monitoring the absorbance at 560 nm due to the reduction of cytochrome *c.* The reaction mixture contained 50 mM phosphate buffer (pH 7.8), 0.1 mM Nitrotetrazolium Blue chloride (NBT), 0.1 mM EDTA and 13.37 mM methionine.

POD activities were determined specifically at 420 nm. The reaction mixture contained 0.4 ml of 100 mM potassium phosphate buffer (pH 6), 0.16 ml of 147 mM H_2_O_2_, 0.32 ml of 5% Pyrogallol and 2.1 ml of DW. The reaction was initiated by adding 20 ul plant extract and after 10 min. The POD activity was determined by following the consumption of H_2_O_2_ (extinction coefficient 39.4 mM^-1^ cm^-1^) at 420 nm for 20 s.

The activity of catalase (CAT) was assayed by monitoring decreases in absorbance at 240 nm due to the decomposition of H_2_O_2_. The reaction mixture contained 670 ul potassium phosphate buffer (pH 7.0), 330 ul H_2_O_2_ and 30 ul of the extract. The CAT activity was determined by following the consumption of H_2_O_2_ (extinction coefficient 39.4 mM^-1^ cm^-1^) at 240 nm for 1 min.

### Lipid peroxidation

Lipid peroxidation was determined as the amount of malondialdehyde (MDA, e = 155 mM ± 1 cm ± 1), a product of lipid peroxidation. 1 ml of saved supernatant (which has also been used to determinate antioxidant enzyme) was mixed with 3 ml reaction buffer including 5% Trichloroacetic acid (TCA) and 0.5% Thiobarbituric acid (TBA) was heated in 100°C water for 15 min, then cooed immediately and centrifuged. The absorbance was monitored at 450, 532 and 600 nm.

## Results

### Confirmation of codA DNA in transgenic potato

**Figure 2:**
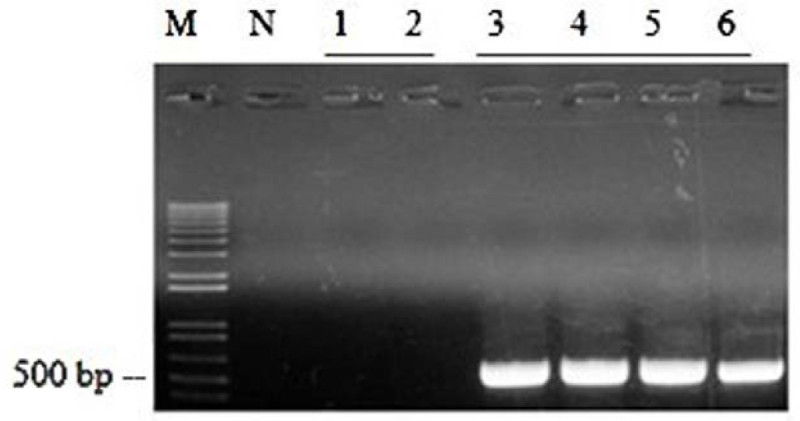
**Development of transgenic potato plants expressing the** ***codA*** **in chloroplasts.** Genomic DNA PCR analysis of the *codA* from transgenic plants. Numbers (3–6) represent independent transgenic lines, M: size marker, number 1–2: non-transgenic plants, N: negative control (water).

As shown in (Figure 2), *codA* does not exist in NT, while the exogenous *codA* gene introduced into transgenic SC plants could be observed. The total length of *codA* was 450 bp as we expected.

### Effect of dehydration pretreatment on GB accumulation and leaf water potential in transgenic and non-transgenic potato

**Figure 3:**
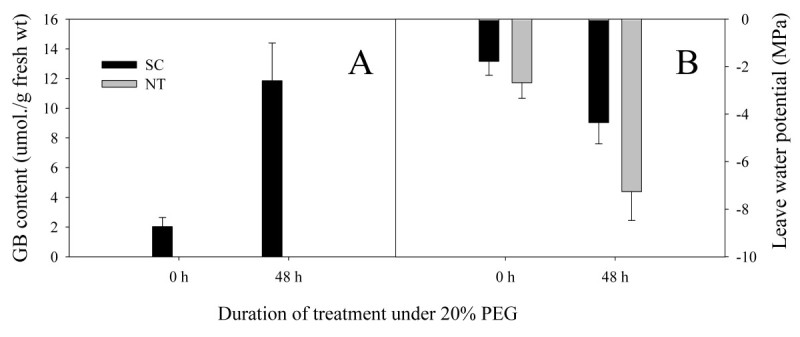
**The determinations of Glycinebetaine contents and leave water potential in potatoes.** GB levels **(A)** and leaf water potential **(B)** in non-transgenic (NT) and transgenic (SC) plants under water stress simulated with 20% PEG. GB was extracted from non-treated and 20% PEG-treated plants. Samples were collected after 48 h of PEG treatment. Data are expressed as the mean ± standard deviation (SD) of five replicates.

To determine whether the expression of *codA* induced the synthesis of GB in the transgenic plants, the GB was analyzed after 0 h and 48 h of 20% PEG stress (Figure 3A). Also, the leaf water potential (LWP) (Figure 3B) of transgenic and non-transgenic potato was determined. At 0 h after stress, no GB accumulated in NT but a slight accumulation was noted in SC. Even at 48 h of stress, no GB was observed in NT, but the amount of GB increased significantly in SC. Leaf water potential of both potato types was significantly reduced from 0 h to 48 h after stress but the LWP of SC remained higher than that of NT.

### Effect of introduced codA gene on enzyme system in transgenic and non-transgenic potato following stress-rehydration treatment

The changes in antioxidant enzyme system (SOD, CAT and POD) of the two potato types under stress-rehydration-treatment are shown in (Table 1, Table 2 and Table 3). Activities of SOD and CAT varied similarly and without significant changes under normal water condition. However, under stress conditions, the SOD and CAT activities in the two potato types increased from DAY 0 to DAY 2; although at DAY 3 the values declined, they were still higher than those on DAY 0 (as an exception, the CAT activities in NT under 20% PEG stress started dropping from DAY 2). From DAY 4 to DAY 5 (rehydration period), SOD and CAT activities in both SC and NT rose again. In general, the SOD and CAT activities of the two potato types were higher under 10% PEG stress than under 20% PEG stress; under the same stress condition, SC showed higher antioxidant enzyme activities than NT.

**Table 1:** **The activities of SOD enzyme in non-transgenic (NT) and transgenic (SC) potatoes during and after stress**

PEG treatments	Potato type	SOD activity (units / mg protein)					
		Stress period				Recovery period	
		DAY 0	DAY 1	DAY 2	DAY 3	DAY 4	DAY5
Control (0% PEG)	SC	16.86±2.05	18.55±3.22	17.1±2.65	17.88±1.85	18.65±1.96	17.3±2.88
	NT	17.07±2.72	18.12±1.75	18.9±3.11	17.6±1.68	16.7±2.84	17.83±2.03
Moderate stress (10% PEG)	SC	18.2±2.21	25.82±4.55	30.15±6.48	23.84±5.14	31.44^*^±4.97	37.28^*^±7.13
	NT	18.21±3.48	21.75±6.54	28.26±5.84	19.52±5.78	20.89±4.22	28.44±7.61
Severe stress (20% PEG)	SC	17.92±1.57	23.18±4.21	26.77^*^±4.33	22.46^*^±6.14	25.06^*^±3.35	29.34±6.92
	NT	16.55±2.41	18.25±3.26	17.25±5.11	11.77±4.68	17.38±3.57	22.21±4.85

Data are expressed as the mean of five replicates. Asterisks (^*^) represent significantly different (P < 0.05) with the NT.

**Table 2:** **The activities of CAT enzyme in non-transgenic (NT) and transgenic (SC) potatoes during and after stress**

PEG treatments	Potato type	CAT activity (units / mg protein)					
		Stress period				Recovery period	
		DAY 0	DAY 1	DAY 2	DAY 3	DAY 4	DAY5
Control (0% PEG)	SC	0.463±0.22	0.472±0.16	0.459±0.19	0.466±0.24	0.461±0.26	0.457±0.22
	NT	0.465±0.20	0.469±0.17	0.463±0.23	0.454±0.19	0.456±0.18	0.465±0.11
Moderate stress (10% PEG)	SC	0.451±0.14	0.814±0.17	0.854±0.26	0.722^*^±0.23	0.907^*^±0.29	1.214^*^±0.42
	NT	0.47±0.15	0.717±0.53	0.653±0.35	0.533±0.24	0.565±0.22	0.896±0.36
Severe stress (20% PEG)	SC	0.459±0.17	0.667±0.26	0.839^*^±0.44	0.676^*^±0.23	0.734^*^±0.47	0.917^*^±0.29
	NT	0.462±0.11	0.508±0.27	0.362±0.24	0.216±0.12	0.369±0.13	0.448±0.18

Data are expressed as the mean of five replicates. Asterisks (^*^) represent significantly different (P < 0.05) with the NT.

**Table 3:** **The activities of POD enzyme in non-transgenic (NT) and transgenic (SC) potatoes during and after stress**

PEG treatments	Potato type	POD activity (units / mg protein)					
		Stress period				Recovery period	
		DAY 0	DAY 1	DAY 2	DAY 3	DAY 4	DAY5
Control (0% PEG)	SC	9.18±0.672	9.22±0.55	8.81±0.84	9.21±0.73	9.11±0.71	8.79±0.57
	NT	8.57±0.98	9.03±0.95	9.52±0.65	9.38±0.42	9.0±0.73	9.25±0.66
Moderate stress (10% PEG)	SC	8.37±0.52	12.86±2.21	18.76±4.47	24.84^*^±5.44	18.6±4.75	9.67^*^±2.48
	NT	9.2±0.86	16.44±3.86	21.3±5.33	28.16±4.16	20.78±4.22	12.07±5.61
Severe stress (20% PEG)	SC	8.84±0.69	15.51±3.26	24.07^*^±4.27	30.38^*^±5.48	25.76±4.85	13.2±3.15
	NT	9.25±1.25	19.83±4.36	28.72±4.21	35.81±6.88	27.39±4.62	15.56±2.48

Data are expressed as the mean of five replicates. Asterisks (^*^) represent significantly different (P < 0.05) with the NT.

Variations in POD activities differed entirely from those for SOD and CAT during the drought stress and rehydration period. Under the four PEG stress conditions, from DAY 0 to DAY 3 POD activities increased progressively before dropping from DAY 4 to DAY 5 in the rehydration period. The POD activities in both potato types were significantly higher under severe than moderate stress; under the same water condition, NT showed a higher POD activity than SC.

### Effect of exogenous codA gene on MDA and chlorophyll contents in transgenic and non-transgenic potato under stress-rehydration treatment

Data for chlorophyll and MDA contents in transgenic and non-transgenic potato are shown in (Tables 4–5). Under normal water condition, both chlorophyll and MDA contents had no significant variation and were within the regular range. Under drought stress, the MDA contents in both potato types increased from DAY 0 to DAY 3 (the period of PEG stress) but reduced in DAY 4 and DAY 5 (the period of hydration).

**Table 4:** **Contents of MDA in non-transgenic (NT) and transgenic (SC) potatoes during and after water stress**

PEG treatments	Potato type	MDA content (mmol.g.FW-1)					
		Stress period				Recovery period	
		DAY 0	DAY 1	DAY 2	DAY 3	DAY 4	DAY5
Control (0% PEG)	SC	4.38±0.58	4.21±0.34	4.25±0.56	4.36±0.34	4.31±0.46	4.28±0.53
	NT	4.35±0.33	4.15±0.26	4.11±0.24	4.43±0.37	4.27±0.41	4.32±0.31
Moderate stress (10% PEG)	SC	4.16±0.61	5.88±1.67	6.92±1.88	8.98±2.17	5.74^*^±1.42	4.81±1.14
	NT	4.24±0.31	6.17±2.54	7.25±1.57	9.02±2.64	7.18±2.47	5.92±1.13
Severe stress (20% PEG)	SC	4.33±0.26	6.36^*^±1.83	9.78^*^±2.46	11.52±2.84	6.29^*^±1.45	5.63^*^±1.57
	NT	4.27±0.27	9.47±2.32	14.24±1.69	13.32±3.68	9.46±2.55	8.38±2.84

Data are expressed as the mean of five replicates. Asterisks (^*^) represent significantly different (P < 0.05) with the NT.

**Table 5:** **Chlorophyll contents in non-transgenic (NT) and transgenic (SC) potatoes during and after water stress**

PEG treatments	Potato type	Chlorophyll content (mg.gFW-1)					
		Stress period				Recovery period	
		DAY 0	DAY 1	DAY 2	DAY 3	DAY 4	DAY5
Control (0% PEG)	SC	1.91±0.26	1.83±0.18	1.86±0.24	1.79±0.23	1.93±0.17	1.87±0.18
	NT	1.84±0.22	1.89±0.17	1.77±0.21	1.9±0.15	1.87±0.24	1.92±0.25
Moderate stress (10% PEG)	SC	1.92±0.28	1.67±0.17	1.45±0.26	1.26^*^±0.34	1.55±0.41	1.83±0.42
	NT	1.85±0.23	1.53±0.35	1.21±0.42	0.97±0.31	1.46±0.39	1.77±0.54
Severe stress (20% PEG)	SC	1.82±0.34	1.49±0.33	1.19±0.56	0.78±0.29	1.32^*^±0.29	1.73^*^±0.38
	NT	1.88±0.19	1.24±0.36	0.95±0.21	0.62±0.18	0.81±0.23	1.37±0.36

Data are expressed as the mean of five replicates. Asterisks (^*^) represent significantly different (P < 0.05) with the NT.

However, the MDA content in NT under 20% PEG stress started dropping earlier at DAY 3 instead of DAY 4. Chlorophyll contents in both plant types decreased from DAY 0 to DAY 3 and leveled off from DAY 4 to DAY 5. In general, the MDA contents in both SC and NT were significantly higher under 20% PEG stress than under 10% PEG stress; under the same stress condition, NT exhibited a higher MDA content than SC. On the contrary, under the four stress conditions, both plant types much higher chlorophyll under 10% PEG stress than under 20% PEG stress; for each stress level, SC showed a significantly higher chlorophyll content than NT.

### Effect of exogenous codA gene on photosynthetic activities in transgenic and non-transgenic potato under stress-rehydration treatment

**Figure 4:**
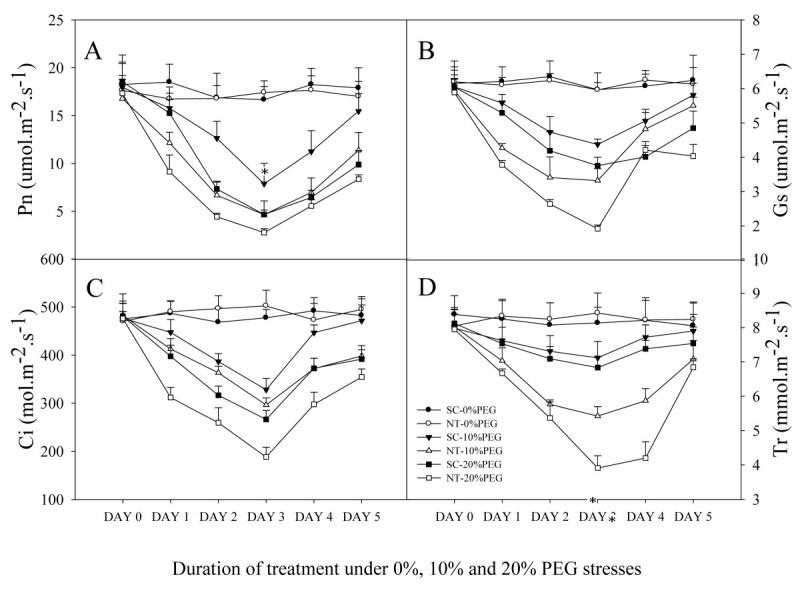
**Changes in photosynthetic parameters of non-transgenic (NT) and transgenic (SC) potatoes during and after water stress. A**: photosynthetic rate (Pn), **B**: Stomatal conductance (Gs), **C**: Intercellular carbon dioxide concentration (Ci), **D**: Transpiration rate. Data are expressed as the mean ± standard deviation (SD) of five replicates.

Data for changes in photosynthetic parameters, including photosynthetic rate (Figure 4A), stomatal conductance (Figure 4B), intercellular carbon dioxide concentration (Figure 4C) and transpiration rate (Figure 4D) of the two potato types due to the introduced gene are showed. There was no apparent variation among photosynthetic indexes of SC and NT under normal water condition. However, all photosynthetic parameters gradually and significantly decreased in both potato types during DAY 0 to DAY 3 under PEG stress, but increased markedly during the period of rehydration from DAY 4 to DAY 5. Throughout the observation period, SC showed higher photosynthetic parameters than NT and 10% PEG was less stressful to plants than 20% PEG.

## Discussion

Glycinebetain is an extremely efficient compatible solute and its presence was strongly associated with enhanced tolerance of plants in stress environments (Rhodes and Hanson 1993). Since some major crops do not produce GB by themselves, studies have been carried out to determine if exogenous application of GB could improve the growth and survival of a wide variety of plants under various stress conditions (Allard et al. 1998), the biosynthesis of GB, and its mechanisms to enhance the tolerate abiotic stress have been deeply reported (Chen and Muata, 2008,2011).

The *codA* gene in this experiment, which was obtained from *Arthrobacter globiformis*, directly converts choline into GB and H_2_O_2_ (Deshnium et al. 1995), and H_2_O_2_, is known to be not only a ROS but also plays many crucial roles as a signalling molecule to induces tolerance to stress (Xiong et al. 2002; Jiang et al. 2012). Kathuria et al. (2009) reported after the transformation of *codA* into rice, the produced H_2_O_2_ could simultaneously activate the response of transgenic plants to stresses. Therefore, GB might not be the only factor that is responsible for the tolerance improvement but the increase of H_2_O_2_ also makes contributions.

From our results, water stress simulated with 20% PEG under the control of SWPA2 promoter, the transgenic potato produced the *codA* gene and accumulated GB while the control plants had neither the *codA* gene nor GB accumulation since potato is a non-GB-accumulating plant. This demonstrated that the gene was successfully transformed and expressed in transgenic potato. When plants were subjected to water stress, the cells consequently lost water. Since one of the key functions of GB is osmoregulation, it could help to maintain the osmotic equilibrium of cells. During pretreatment, the overwhelming advantage of leaf water potential in SC relative to NT was based on the accumulation of GB.

When plants were subjected to water stress, reactive oxygen species (ROS) accumulated; at the same time the antioxidant system, especially SOD, the most important antioxidant enzyme was induced to scavenge the newly produced ROS. To determine the influence of accumulated GB on the antioxidant enzyme system and photosynthetic system of transgenic potato during water stress and recovery stage, we measured the daily activities of SOD, POD and CAT, as well as photosynthetic parameters of two plant types.

The changes in SOD and CAT activities in the two plant types followed a similar trend under water stress. The activities of both enzymes increased across stress treatments from DAY 0 to DAY 2. At DAY 3, the activities slightly reduced compared to DAY 2 but were still higher than DAY 0 because the amount of accumulated ROS exceeded the scavenging ability of antioxidant enzymes, hence, plant cells might been hurt. Ultimately from DAY 4 to DAY 5 (rehydration period) when plants were provided with normal water condition, the injured plant cells regained their structure, function and the enzyme activities rose again. Also, the activities of SOD and CAT increased more significantly in SC than NT during rehydration stage. Therefore, after stress, SC was more able to eliminate ROS and protect plants.

The POD activities of two plant types changed in a quite different way compared with SOD and CAT during the whole treatment. They gradually increased from DAY 0 to DAY 3 under water stress and reduced from DAY 4 to DAY 5 in the recovery stage. This result can be explained by the dual functional role of POD in plants. On the one hand, it can express a protective effect as a member of the scavenging enzyme system that removes H_2_O_2_ at the earlier stage of stress or aging; on the other hand, it can be also express injurious effects at the later stage of stress or aging, prompting the generation of reactive oxygen species, degradation of chlorophyll and peroxidation of membrane lipids which are products as well as indices of aging or stress. The main role of POD is generally considered to be the later one (Zhang and Kirkham 1994). Thus, due to the water stress, especially under the severe drought treatment (20% PEG), POD activities increased markedly in both potato types. During the recovery period, the antioxidant system and other physiological reactions regained their abilities resulting to removal of ROS and reduction of POD activities. In contrast to SOD and CAT, the POD activity was higher in NT than in SC, possibly because the promoter used in this experiment was derived from moderating clips of POD enzyme (SWPA2) in sweet potato. Exogenous promoters can cause transcriptional gene silencing of endogenous unlinked homologous promoters (Mette et al. 1999). Therefore, the expression of endogenous POD in SC was effected by the introduced *CodA* expressing construct which were regulated by the SWPA2 promoter. The expression of the endogenous promoter of POD enzyme might be suppressed by the inserted exogenous promoter and the activity of POD was inhibited to some level in SC. The SOD and CAT activities in SC were markedly superior to NT during the whole experiment. Hence, it could be suggested that the GB produced by the introduced *codA* gene in potato could protect its antioxidant system and enhance its drought resistance.

The protection of photosynthetic system against photodamage Because GB could internally stabilize the photosynthetic apparatus, improve the practical efficiency of photosynthetic system II and cell osmotic equilibrium (Papageorgiou and Murata 1995), the transgenic potato with extra GB exhibited much higher photosynthetic parameters than NT. For example, at DAY 1 of water stress with 10% PEG, the photosynthetic rate (Pn) and stomatal conductance (Gs) in SC dropped only slightly compared with control treatment. In addition, the intercellular carbon dioxide concentration (Ci) and transpiration rate (Tr) were higher in SC than in NT indicating that these parameters were directly related to Pn and Gs. During the recovery stage, as GB could protect the repair machinery of PS (Ohnishi and Murata, 2006; Murata et al. 2007), the photosynthetic parameters of SC recovered more efficiently than NT after stress. Moreover, the higher leaf water potential in SC (which has been proved in pretreatment) sustained a higher Tr. The MDA content was higher in NT than in SC while chlorophyll content showed an opposite trend in this experiment. This demonstrated that the GB produced by introduced *codA* gene in SC could prevent membrane lipid peroxidation and degradation of chlorophyll caused by stress.

## Conclusion

In conclusion, the exposure of transgenic potato to four-day-stress (with 10% and 20% PEG) and two-day-recovery periods favoured the accumulation of GB and H_2_O_2_ by *codA* transgene; consequently the transgenic potato showed stronger antioxidant enzyme ability, more efficient photosynthetic system, higher leaf water potential and chlorophyll content and lower MDA content. It also showed better recovery from stress than the non-transgenic potato. The exogenous *codA* gene provided potato a stronger drought resistance and recovery ability.

## Authors’ original submitted files for images

Below are the links to the authors’ original submitted files for images. Authors’ original file for figure 1 Authors’ original file for figure 2 Authors’ original file for figure 3 Authors’ original file for figure 4

## Abbreviations

COD: Choline oxidase
CAT: Catalase
GB: Glycine betaine
MDA: Malondialdehyde
PEG: Polyethylene glycol
POD: Peroxidase
ROS: Reactive oxygen species
SOD: Superoxide dismutase.
