# Supplementary figures and images for: Enhanced tolerance of transgenic potato plants expressing choline oxidase in chloroplasts against water stress
Source: Bot Stud. 2013 Sep 3;54:30. doi: 10.1186/1999-3110-54-30 (PMC5432851; doi:10.1186/1999-3110-54-30)

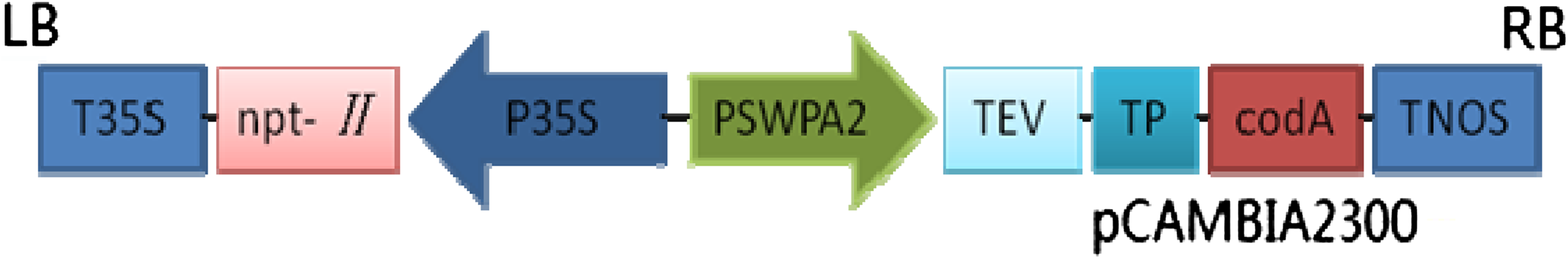

Supplement: Supplementary file 1 — Authors’ original file for figure 1 [file 40529_2012_30_MOESM1_ESM.tif]

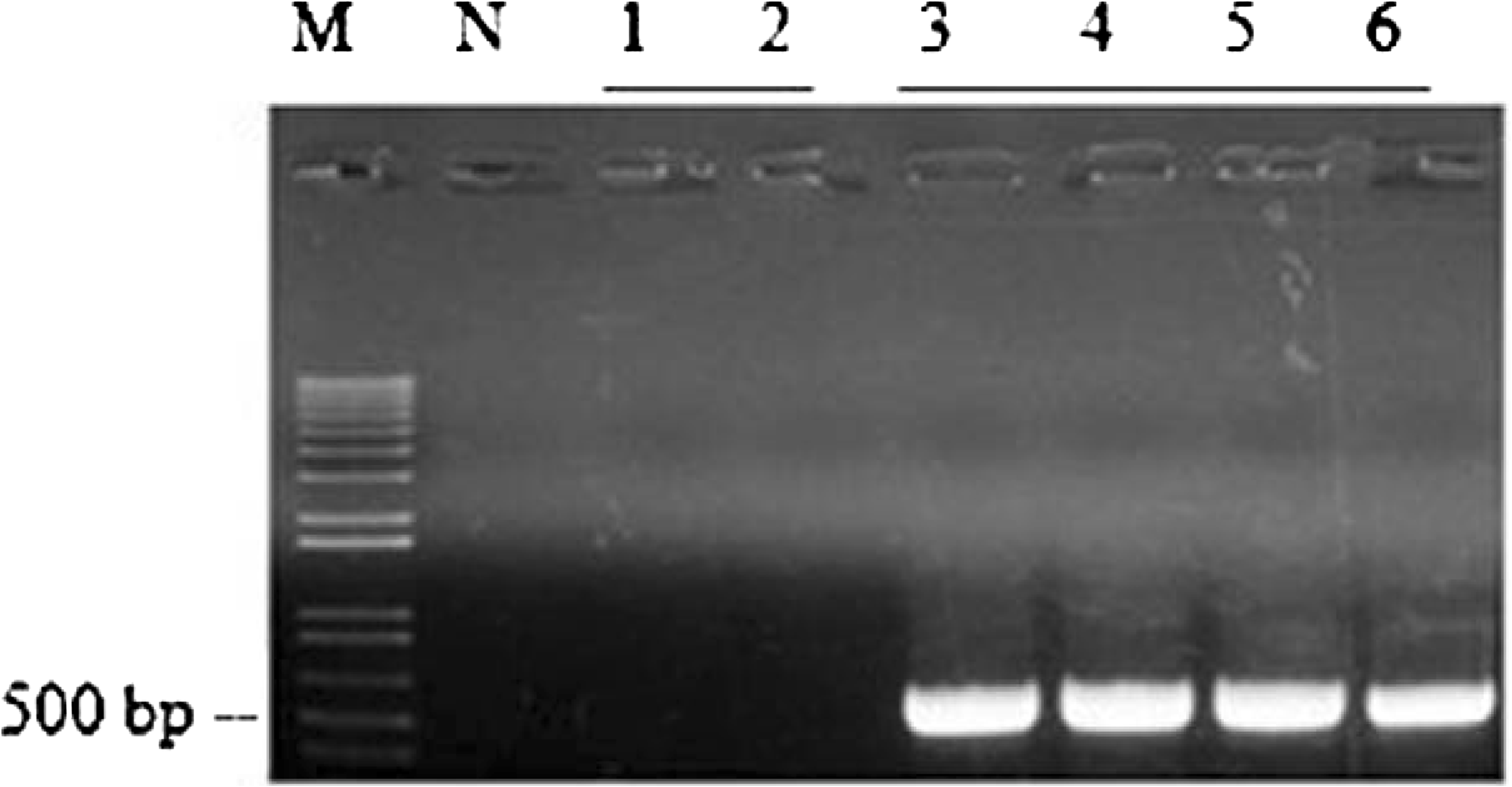

Supplement: Supplementary file 2 — Authors’ original file for figure 2 [file 40529_2012_30_MOESM2_ESM.tif]

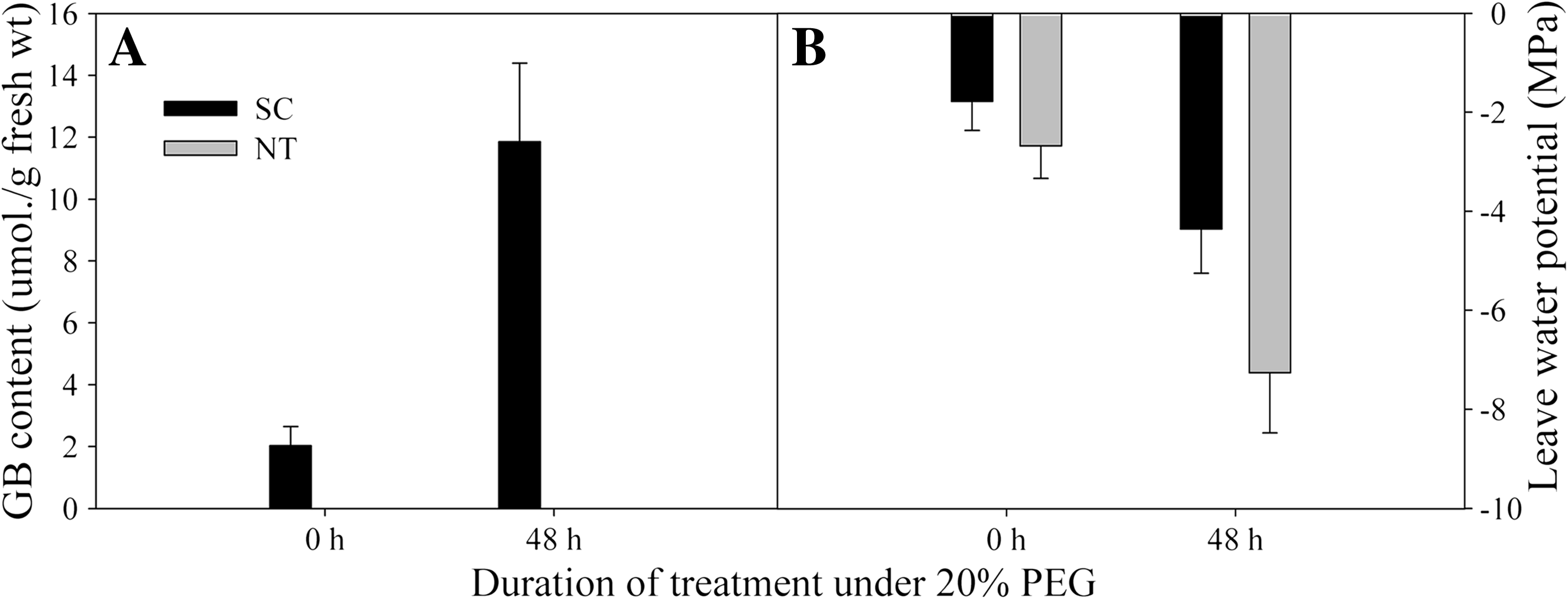

Supplement: Supplementary file 3 — Authors’ original file for figure 3 [file 40529_2012_30_MOESM3_ESM.tif]

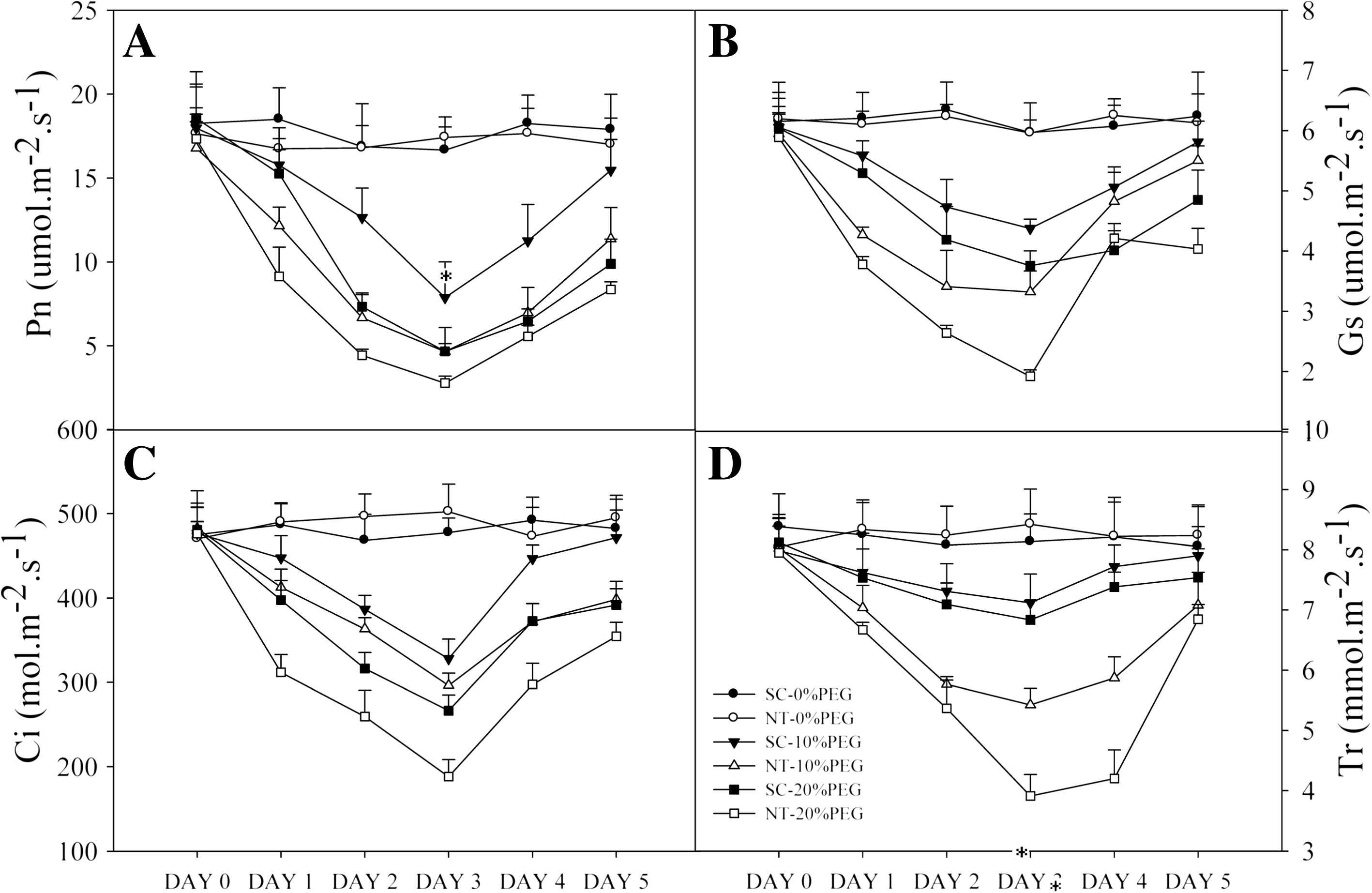

Supplement: Supplementary file 4 — Authors’ original file for figure 4 [file 40529_2012_30_MOESM4_ESM.tif]
